# Assessment of Muscle-Specific Strength in Oncology Patients: Anthropometry as a Reliable Alternative to DXA

**DOI:** 10.3390/life15081300

**Published:** 2025-08-15

**Authors:** Blanca Alabadi, Sandra Amores, Miriam Moriana, Ning Yun Wu Xiong, Katherine García-Malpartida, José Antonio Pedrón, Clàudia Monrós, Sergio Martínez-Hervás, José T. Real, Miguel Civera

**Affiliations:** 1 Service of Endocrinology and Nutrition, Hospital Clínico Universitario of Valencia, 46010 Valencia, Spain; balabadi@incliva.es (B.A.); samores@incliva.es (S.A.); mmoriana.nutricion@gmail.com (M.M.); nywx.224@hotmail.com (N.Y.W.X.); kathe.garciamalpartida@gmail.com (K.G.-M.); pedron_josbar@gva.es (J.A.P.); claudiamonrosperis@gmail.com (C.M.); jtreal@uv.es (J.T.R.); mi.civeraa@comv.es (M.C.); 2 INCLIVA Biomedical Research Institute, 46010 Valencia, Spain; 3 CIBER de Diabetes y Enfermedades Metabólicas Asociadas (CIBERDEM), 28029 Madrid, Spain; 4 School of Health Sciences, Universidad Cardenal Herrera—CEU, CEU Universities, Calle Grecia 31, 12006 Castellón, Spain; 5 Department of Medicine, University of Valencia, 46010 Valencia, Spain

**Keywords:** muscle-specific strength, sarcopenia, cancer, body composition, dual-energy X-ray absorptiometry, anthropometry

## Abstract

**Background**: Muscle-specific strength (MSS), defined as muscle strength relative to muscle mass, is a key indicator in the assessment of sarcopenia and functional status, especially in patients with cancer. Dual-energy X-ray absorptiometry (DXA) is the reference method for estimating muscle mass, but its limited accessibility hinders routine use. This study aimed to evaluate the correlation between DXA-based MSS and MSS estimated through more accessible techniques. **Methods**: A cross-sectional study was conducted in 205 adult oncology outpatients. Muscle strength was assessed by handgrip dynamometry, and muscle mass was estimated using DXA, bioimpedance (BIA), ultrasound, and anthropometry. MSS was calculated by dividing grip strength by each muscle mass parameter. **Results**: MSS calculated with BIA, body weight, and calf circumference showed very strong correlations with MSS-DXA (r > 0.90). Ultrasound-derived MSS showed only a moderate correlation (r = 0.55; *p* < 0.01). Similar patterns were observed in both men and women. **Conclusions**: BIA and anthropometric methods, particularly those using body weight and calf circumference, are reliable and accessible alternatives to DXA for estimating MSS in oncology patients. These tools may help improve the identification and monitoring of sarcopenia in clinical settings with limited resources.

## 1. Introduction

The study of muscle tissue and its associated diseases has gained importance in recent decades due to its impact on health status and quality of life [1,2]. Among these conditions, sarcopenia has garnered increasing attention, particularly in vulnerable populations such as cancer patients. In this group, the loss of muscle mass and function is common due to the disease itself, oncologic treatments, and metabolic disturbances, which may negatively impact both quality of life and patient prognosis [3,4]. Currently, the mainstay of treatment for sarcopenia includes individualized physical exercise—especially resistance training—and a healthy diet that ensures an adequate protein intake, both of which are key to maintaining or recovering muscle mass and strength [5].

Currently, in our setting, the most used diagnostic algorithm for sarcopenia is the one established by the European Working Group on Sarcopenia in Older People (EWGSOP), which considers three key components: muscle strength, muscle mass, and physical performance [6]. Despite advances, there remains a lack of international consensus on the diagnostic criteria for sarcopenia, prompting the creation of the Global Leadership Initiative on Sarcopenia (GLIS) between 2019 and 2021, with the aim of establishing unified criteria for assessment. Through a Delphi study, consensus was reached on several key aspects, highlighting muscle mass (89.4%), muscle strength (94.1%), and muscle-specific strength (80.8%) as fundamental components of sarcopenia, as well as physical impairment as one of its consequences [7].

**Figure 1:**
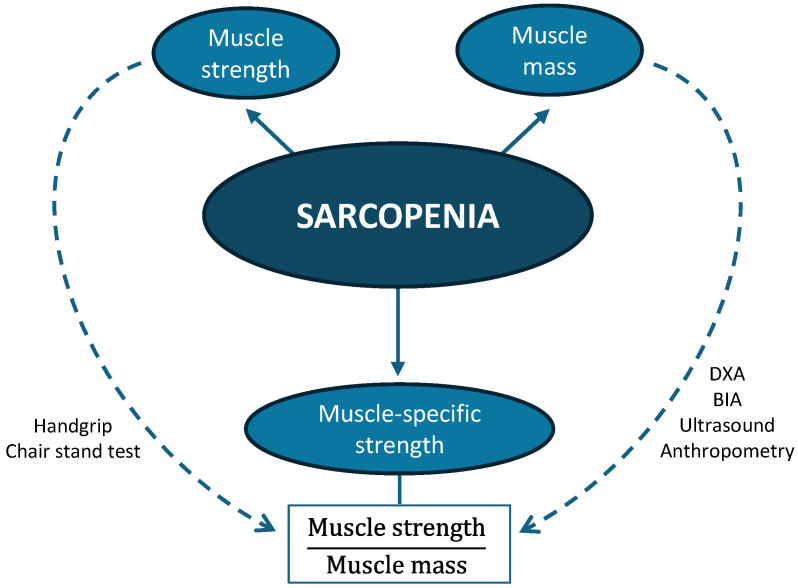
Key components of sarcopenia and muscle-specific strength evaluation methods.

Muscle-specific strength (MSS), as illustrated in Figure 1, evaluates muscle function and is defined as the amount of strength standardized by the amount of muscle tissue [8]. MSS has emerged as a relevant indicator of functional muscle quality, with the potential to offer a more accurate assessment than absolute strength—which does not account for body volume—particularly in populations with obesity or cancer [9,10]. In oncology patients, MSS has been shown to be a significant predictor of survival and postoperative complications [11,12].

Measuring MSS requires combining techniques to assess both strength and body composition [8]. Dual-energy X-ray absorptiometry (DXA) is considered the gold standard for measuring muscle mass; however, its limited availability and high cost restrict its use in many clinical settings [12]. Therefore, it is necessary to explore more accessible and cost-effective alternatives such as bioelectrical impedance analysis (BIA), nutritional ultrasound, and anthropometry, which may provide valid and useful estimates for calculating MSS.

Although the clinical utility of these more accessible methods has been suggested in other populations, their validity and correlation with the gold standard in oncology patients requires rigorous analysis [13,14,15]. Identifying accurate alternative techniques would allow resource-limited centers to improve the functional assessment of patients, optimizing diagnosis, monitoring, and intervention in oncologic sarcopenia.

Therefore, the present study aims to evaluate the correlation between muscle-specific strength adjusted by DXA and MSS calculated through bioimpedance, ultrasound, and anthropometry in a population of oncology patients. This research seeks to provide practical and applicable solutions to support a comprehensive approach to sarcopenia in clinical settings with varying technological capacities.

## 2. Materials and Methods

### 2.1. Participants

A total of 205 patients over 18 years of age with active oncological disease who attended the Body Composition Unit (a research platform of INCLIVA, Research Institute of the Hospital Clínico of Valencia) on an outpatient basis were included.

Patients were excluded if they were unable to maintain the proper position for image acquisition and/or had metal implants or prostheses, pacemakers, surgical clips, or radiopaque tubes or catheters. Additionally, patients who had received iodinated or barium contrast agents for other imaging tests in the previous 7 days had their DXA rescheduled one week later.

### 2.2. Anthropometric Variables

Anthropometric parameters were assessed using standardized procedures: weight (kg), height (m), body mass index (BMI) (kg/m^2^), calf circumference at its most prominent point (cm), mid-arm circumference between the acromion and olecranon (cm), and triceps skinfold measured at the midline between the acromion and olecranon (mm).

Circumferences were measured using a flexible, non-extensible ergonomic Cescorf^®^ (Porto Alegre, Brazil) tape, and the triceps skinfold was measured with a Holtain^®^ LTD (Crymych, UK) caliper set at a pressure of 10 g/mm^2^. All measurements were taken by the same investigator.

Mid-arm muscle area (MMA) was calculated using mid-arm circumference (MAC) and triceps skinfold (TS) according to the following formula [16]:

(1) $$\mathrm{MMA}=\frac{{\mathrm{MAC}}^{2}-{\left(3.14\times \mathrm{TS}\right)}^{2}}{4\times 3.14}$$

### 2.3. Body Composition Assessment

Body composition variables were assessed after 12 h of fasting and no physical activity for at least 8 h.

First, bioimpedance analysis was performed using two devices, a single-frequency (BIAsf) device (NutriLab, Akern, Pontassieve, Italy) and a multi-frequency (BIAmf) device (INBODY 770, Seoul, Republic of Korea), following standardized methodology [17]. In both cases, appendicular skeletal muscle mass was estimated using the Sergi equation [18].

Second, a DXA scan (Horizon Wi, Hologic, Marlborough, MA, USA) was used to assess body composition based on X-ray attenuation properties of different body compartments [19]. The equipment was calibrated daily using a spine phantom to ensure measurement accuracy. Lastly, a muscle ultrasound was performed. To ensure reliable and consistent measurements, all ultrasounds were performed by a trained investigator. A Mindray Z50 (Shenzhen, China) ultrasound machine was used with MSK settings, a linear transducer, and a 10 MHz frequency.

The ultrasound (US) was conducted with the patient in the supine position, with the knee fully extended and after 5 min of rest. The rectus femoris of the dominant leg was assessed, and measurements were taken between the anterior superior iliac spine and the proximal end of the patella, at the proximal 30% point above the superior edge of the patella. The probe was placed perpendicular to the thigh following standardized methods [20,21]. Each measurement was repeated three times, and the average was used for analysis.

### 2.4. Muscle-Specific Strength

Muscle strength was evaluated using handgrip strength of the dominant hand with a digital dynamometer (Jamar Plus+, Patterson Medical, Warrenville, IL, USA), following the Southampton protocol [22]. The highest value from three measurements taken one minute apart was recorded in kilograms.

Muscle-specific strength was calculated by dividing the handgrip strength (kg) by different parameters, as follows [8]:

- MSS/height: handgrip strength divided by height (m);
- MSS/weight: handgrip strength divided by body weight (kg);
- MSS/BMI: handgrip strength divided by BMI;
- MSS/calf: handgrip strength divided by calf circumference (cm);
- MSS/MMA: handgrip strength divided by mid-arm muscle area (cm^2^);
- MSS/US: handgrip strength divided by rectus femoris area (cm^2^);
- MSS/BIAsf: handgrip strength divided by appendicular muscle mass from single-frequency BIA (kg);
- MSS/BIAmf: handgrip strength divided by appendicular muscle mass from multi-frequency BIA (kg);
- MSS/DXA: handgrip strength divided by appendicular lean mass measured by DXA (kg).

### 2.5. Statistical Analysis

Data analysis was performed using SPSS version 27 for iOS (SPSS, Chicago, IL, USA). All variables are expressed as mean ± standard deviation. A *p*-value < 0.05 was considered statistically significant.

Descriptive analyses were used to characterize the study cohort. The Shapiro–Wilk test was applied to assess normal distribution. Differences by sex were analyzed using Student’s *t*-test or the Mann–Whitney U test, as appropriate. Levene’s test was used to assess homogeneity of variances.

Spearman correlation was used to assess associations between variables, as not all followed a normal distribution. Correlation strength was interpreted using the absolute value of r: moderate: r ≤ 0.4; strong: r ≤ 0.6; and very strong: r ≤ 0.8 [23].

Scatterplots were generated to visualize the relationships between MSS/DXA and MSS estimated by BIAsf and BIAmf, stratified by sex, with different colors used for men and women.

## 3. Results

A total of 205 patients were included, 133 men and 72 women, with a mean age of 62 years. Clinical, anthropometric, body composition, and functional data for the overall cohort and by sex are shown in Table 1. Table 2 shows muscle-specific strength calculated using different parameters.

As expected, men showed a significantly higher amount of muscle mass than women, even with similar BMI values, according to measurements from anthropometry, ultrasound, bioimpedance, and DXA. Likewise, men had significantly higher maximal handgrip strength and muscle-specific strength across all adjusted parameters.

Table 3 presents the correlations between MSS/DXA and the other evaluated parameters, considering both the overall cohort and the analyses stratified by sex.

A very strong correlation was observed between MSS/DXA and MSS/weight, MSS/BIAsf, and MSS/BIAmf, both in the overall analysis and in the sex-stratified analyses.

MSS/height, MSS/BMI, MSS/calf, and MSS/MMA showed a strong correlation with MSS/DXA in the full cohort. When analyzing men only, the strength of the correlation remained consistent across all variables, except for MSS/calf, which showed a very strong correlation. Among women, these variables also exhibited very strong correlations, except for MSS/height, which remained strong.

Finally, the only moderate correlation identified was between MSS/DXA and MSS/US, both in the total sample and in the analyses stratified by sex.

**Figure 2:**
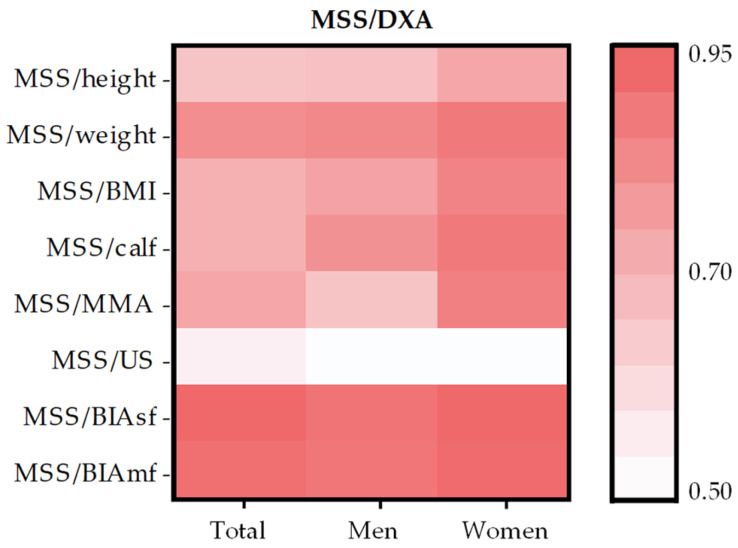
Correlation matrix between MSS/DXA and MSS calculated from other variables.

**Figure 3:**
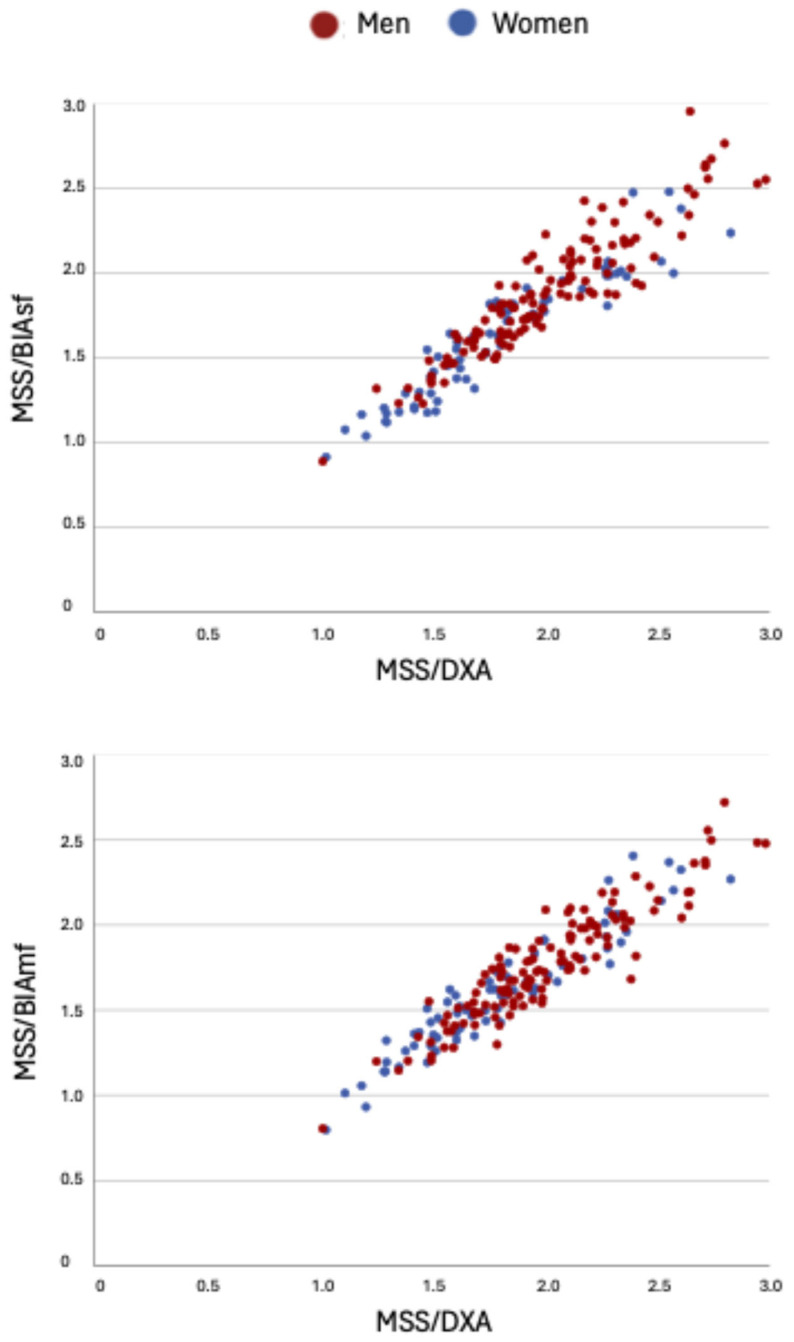
Scatterplots of MSS/DXA versus MSS/BIAsf and MSS/BIAmf, stratified by sex.

The same data are visually represented in Figure 2 using a color-coded correlation matrix, while Figure 3 shows scatterplots illustrating the relationships between MSS/DXA and MSS estimated by BIAsf and BIAmf.

## 4. Discussions

This study assessed the correlation between muscle-specific strength obtained by DXA—the reference standard—and its estimates derived from more accessible techniques such as anthropometry, bioimpedance, and ultrasound in an oncology population. Our findings reveal very strong correlations between MSS/DXA and MSS calculated using bioimpedance and various anthropometric variables, suggesting that these alternatives can serve as valid tools for functional muscle assessment in resource-limited settings. From a clinical nutrition perspective, these correlations are relevant because they enable dietitians and physicians to monitor the effect of tailored nutritional interventions—especially protein-focused strategies—without relying on costly imaging.

The cohort had a mean BMI of 25.5 kg/m^2^ and a mean age of 62 years, with no significant age or BMI differences between men and women. As expected, body-composition and muscle-function variables differed by sex: men showed higher muscle mass on every technique—anthropometry, muscle ultrasound, single- and multi-frequency BIA, and DXA. These results align with previous studies in similar populations, reinforcing existing evidence of sex-based differences in body composition and muscle function [24].

Likewise, regardless of the adjustment technique, MSS was consistently higher in men than in women, implying that, for a comparable amount of muscle, men generate greater force. Prior work has consistently shown that greater adipose infiltration within or between muscle fibers and fascia is linked to lower muscle strength [25]. Women—even at similar body weight and muscle mass to men—tend to accumulate more intramuscular lipids due to hormonal factors such as estrogen influence [26,27], which could explain their lower MSS.

Oncologic disease can markedly accelerate muscle deterioration owing to chemotherapy, radiotherapy, surgical procedures, anorexia, reduced physical activity, systemic inflammation, and tumor-derived catabolic mediators. In this context, evaluating muscle mass and function becomes even more critical than in the general population. A recent study demonstrated that MSS independently associates with postoperative complications and survival after curative colectomy for colorectal cancer [10,11]. Likewise, adding MSS measurement to the sarcopenia definition enhances its prognostic value for postoperative outcomes in cancer patients [28], supporting the GLIS consensus that low MSS is a core component of sarcopenia [7]. Similar results have been reported in general in-patient populations, where Ferri Burgel et al. showed MSS to be an independent predictor of six-month mortality [13].

Notably, international guidelines (e.g., European Society for Clinical Nutrition and Metabolism, ESPEN, and American Society of Clinical Oncology, ASCO) recommend combining resistance exercise with a protein intake of 1.2–1.5 g/kg/day in oncology patients to preserve lean mass and improve functional outcomes; thus, easily obtainable MSS estimates could help monitor response to these nutrition-and-exercise prescriptions in routine care [29].

MSS also has clinical relevance beyond cancer. In obesity, excess adiposity can mask muscle weakness when absolute strength values are used. Muscle mass may be preserved—or even increased—but functional capacity can still be impaired due to low MSS. Factors such as myosteatosis, chronic inflammation, and mitochondrial dysfunction compromise muscle contractility, so focusing solely on muscle mass without considering quality may underestimate functional and metabolic risk in individuals with obesity [8]. Several studies evaluating body composition and physical function in people with obesity undergoing weight-loss interventions—ranging from GLP-1 receptor agonists to bariatric surgery—have detected significant muscle-mass loss. Nevertheless, physical-function scores improve, even with substantial weight reduction, indirectly suggesting that MSS may improve after treatment despite the decrease in muscle mass [30,31,32]. This reinforces the importance of assessing muscle quality rather than quantity alone when designing nutritional and pharmacological weight-loss strategies.

Most studies supporting MSS for prognostic or clinical applications rely on high-precision imaging such as computed tomography (CT) or DXA. These methods are costly, less accessible, and complex, limiting their routine use in many centers. Our results indicate that although bioimpedance and anthropometry are less precise for muscle-mass assessment, they offer excellent alternatives for determining MSS because they show very strong correlations with DXA-derived MSS, the accepted gold standard. In this regard, bioimpedance—both single- and multi-frequency—stands out as the best DXA substitute in both sexes. Weight and calf circumference follow closely and are even simpler to obtain, highlighting anthropometry’s utility as an accessible, practical tool for estimating MSS. Importantly, these field methods can be implemented by clinical nutrition teams during routine assessments, facilitating rapid feedback on the effectiveness of dietary protein prescriptions or oral nutritional supplements.

The lower correlation observed between MSS/DXA and MSS/US could be attributed to the fact that ultrasound does not quantify total body muscle mass but is limited to the assessment of the rectus femoris muscle. Although calf circumference also represents a regional measure, ultrasound is subject to greater interobserver variability, as its accuracy largely depends on the evaluator [33]. Assessments in this cohort were performed by two different evaluators, which, despite their sufficient experience, may explain the moderate correlation observed.

Some authors have already explored MSS assessment via anthropometry, showing that adjustments using MMA, mid-arm circumference, or calf circumference adjusted for BMI associate inversely with adverse outcomes during hospitalization and at six months post-discharge [13]. Recently, cut-off points have also been proposed for MSS adjusted by body weight, BMI, stature, and appendicular skeletal muscle mass estimated by bioimpedance [15].

To our knowledge, no previous study had examined these parameters’ correlation with the reference technique. Our data help fill the gap identified by Vieira et al., who called for validating more affordable MSS methods such as calf-circumference measurement [9].

Key strengths of this study include its sizeable sample and the exclusive inclusion of patients with active cancer, enhancing applicability to this clinical population. Concurrent use of multiple assessment techniques provides a comprehensive, comparative approach to MSS, strengthening internal validity. All measurements were performed by trained personnel using standardized protocols, minimizing inter-observer variability and boosting data reliability.

Limitations include the potential lack of generalizability to other clinical groups or to patients with more advanced or functionally impaired disease, as well as the fact that the type and stage of cancer were not recorded—factors that may independently influence muscle mass and function. The cross-sectional design precludes assessing the long-term predictive value of alternative techniques versus DXA. Despite staff training, measurements such as anthropometry and ultrasound may still entail technical variability, especially in obese or cachectic patients. Furthermore, we acknowledge that inter-rater reliability for ultrasound measurements was not formally assessed, which represents a methodological limitation. Additionally, we did not collect detailed dietary intake data or biomarkers of nutritional status, which could have provided insight into the interaction between diet quality, protein adequacy, and MSS.

These findings offer valuable insight into the feasibility and clinical applicability of anthropometric methods for estimating MSS in cancer patients. In many healthcare settings—especially in resource-limited or outpatient oncology environments—access to DXA is restricted or unavailable. Our results suggest that MSS estimation using simple anthropometric measurements, such as body weight and calf circumference, or accessible tools like BIA, can serve as reliable alternatives to DXA. This enables healthcare professionals, including dietitians, oncologists, and rehabilitation specialists, to monitor changes in muscle quality over time, evaluate the effectiveness of nutritional or exercise interventions, and potentially improve patient outcomes through earlier identification of sarcopenia. By facilitating routine functional assessment, these methods may help personalize treatment strategies and optimize supportive care in oncology.

Nonetheless, multicenter studies with larger, more diverse populations and longitudinal designs are needed to confirm our results, examine temporal changes in MSS, and determine their impact on clinically relevant outcomes, thereby formulating robust and widely applicable recommendations.

## 5. Conclusions

Muscle-specific strength estimated through anthropometry shows a very strong correlation with the DXA-derived measurement in oncology patients. In particular, MSS adjusted by calf circumference and body weight represents a reliable and accessible alternative for assessing muscle function in clinical settings with limited resources. These findings support the use of cost-effective and practical methods that facilitate accurate identification and monitoring of sarcopenia in this vulnerable population, thereby expanding their applicability across a variety of healthcare contexts.

## Figures and Tables

**Table 1:** Characteristics of the patients included in the study.

	Total (*n* = 205)	Men (*n* = 133)	Women (*n* = 72)
Age (years)	62.0 ± 11.8	62.5 ± 11.8	61.1 ± 11.8
Weight (kg)	71.9 ± 15.4	75.9 ± 14.8	64.5 ± 13.7 *
Height (m)	1.67 ± 0.10	1.72 ± 0.08	1.59 ± 0.06 *
Body mass index (kg/m^2^)	25.5 ± 4.6	25.5 ± 4.2	25.4 ± 5.2
Calf circumference (cm)	35.3 ± 4.0	25.7 ± 4.0	34.5 ± 3.8 *
Mid-upper-arm circumference (cm)	29.8 ± 4.1	30.3 ± 4.1	29.0 ± 4.0 *
Triceps skinfold (mm)	16.3 ± 8.4	13.7 ± 7.9	21.6 ± 6.7 *
Mid-arm muscle area (cm^2^)	48.7 ± 14.1	54.3 ± 13.5	37.3 ± 6.5 *
Rectus femoris area (cm^2^)	4.48 ± 1.60	4.88 ± 1.66	3.72 ± 1.16 *
Appendicular muscle mass by BIAsf (kg)	19.6 ± 4.5	21.8 ± 3.7	15.6 ± 2.6 *
Appendicular muscle mass by BIAmf (kg)	20.7 ± 5.1	23.4 ± 4.1	16.0 ± 2.7 *
Appendicular lean mass by DXA (kg)	18.2 ± 4.4	20.4 ± 3.6	14.2 ± 2.4 *
Maximum muscle strength (kg)	35.3 ± 11.0	40.5 ± 9.4	25.3 ± 5.6 *

Data are shown as average ± standard deviation. * *p* < 0.05 between women and men.

**Table 2:** Muscle-specific strength calculated by different parameters.

	Total (*n* = 205)	Standard Error of Estimate	Men (*n* = 133)	Women (*n* = 72)
MSS/height	20.8 ± 5.8	0.41	23.5 ± 5.0	15.8 ± 3.3 *
MSS/weight	0.49 ± 0.14	0.01	0.54 ± 0.13	0.40 ± 0.10 *
MSS/BMI	1.41 ± 0.47	0.03	1.62 ± 0.42	1.02 ± 0.27 *
MSS/calf	1.00 ± 0.28	0.02	1.13 ± 0.23	0.74 ± 0.16 *
MSS/MMA	0.74 ± 0.19	0.01	0.77 ± 0.19	0.68 ± 0.17 *
MSS/US	8.37 ± 2.75	0.19	8.95 ± 2.81	7.30 ± 2.28 *
MSS/BIAsf	1.79 ± 0.38	0.03	1.88 ± 0.36	1.63 ± 0.36 *
MSS/BIAmf	1.71 ± 0.35	0.02	1.77 ± 0.33	1.60 ± 0.35 *
MSS/DXA	1.94 ± 0.39	0.03	2.01 ± 0.36	1.80 ± 0.40 *

Data are shown as average ± standard deviation. * *p* < 0.05 between women and men.

**Table 3:** Correlations between MSS/DXA and MSS calculated from other variables.

	Total (*n* = 205)	Men (*n* = 133)	Women (*n* = 72)
	MSS/DXA		
MSS/height	0.68 *	0.69 *	0.77 *
MSS/weight	0.84 *	0.86 *	0.90 *
MSS/BMI	0.74 *	0.78 *	0.87 *
MSS/calf	0.74 *	0.83 *	0.90 *
MSS/MMA	0.77 *	0.68 *	0.88 *
MSS/US	0.55 *	0.51 *	0.51 *
MSS/BIAsf	0.95 *	0.92 *	0.95 *
MSS/BIAmf	0.93 *	0.91 *	0.94 *

* *p* < 0.01.

## Data Availability

All data are available from the corresponding author upon reasonable request.

## Abbreviations

The following abbreviations are used in this manuscript:

BIA	Bioelectrical impedance analysis
BIAsf	Single-frequency bioelectrical impedance analysis
BIAmf	Multi-frequency bioelectrical impedance analysis
BMI	Body mass index
CT	Computed tomography
DXA	Dual-energy X-ray absorptiometry
EWGSOP	European Working Group on Sarcopenia in Older People
GLIS	Global Leadership Initiative on Sarcopenia
GLP-1	Glucagon-like peptide-1
MAC	Mid-arm circumference
MMA	Mid-arm muscle area
MSS	Muscle-specific strength
US	Ultrasound
TS	Triceps skinfold
